# Epidemic Intelligence Service Application Deadline — August 15, 2014

**Published:** 2014-06-20

Applications are being accepted for CDC’s July 2015–June 2017 Epidemic Intelligence Service (EIS) program. EIS is a 2-year, postgraduate program of service and on-the-job training for health professionals interested in the practice of epidemiology. Each year, EIS selects approximately 80 persons from applicants around the world and provides them with opportunities to gain hands-on experience in epidemiology at CDC or at state or local health departments. EIS officers, often called CDC’s “disease detectives,” have gone on to occupy leadership positions at CDC and other public health agencies nationally and internationally. However, the experience also is useful for health professionals who want to gain a population health perspective.

Persons with a strong interest in applied epidemiology who meet at least one of the following qualifications may apply to EIS: 1) a doctoral-level scientist with a degree in behavioral science, biostatistics, epidemiology, informatics, or nutrition; 2) a health professional such as a veterinarian, nurse (with a bachelor of science or master of science in nursing degree), or a dentist with a master of public health degree or the equivalent; or 3) a physician with at least 1 year of clinical training.

For most positions in the EIS class, U.S. citizenship or permanent residency and an active, unrestricted U.S. license to practice a clinical specialty are requirements. Non-U.S. citizens eligible for a J-1 visa may apply, but a limited number are selected. Information regarding the EIS online application and program details is available at http://www.cdc.gov/eis/applynow.html; by telephone (404-498-6110); or by e-mail (eis@cdc.gov).

